# Risk factors of neurocognitive disorders and dementia in older people in rural districts of Eastern Uganda

**DOI:** 10.1002/alz70860_103803

**Published:** 2025-12-23

**Authors:** Monica M Diaz, Stephen Ojiambo Wandera, Marleny Nolasco, Shafiq Kawooya, Lodrick Wabwire Odo, Noeline Nakasujja

**Affiliations:** ^1^ University of North Carolina at Chapel Hill School of Medicine, Chapel Hill, NC, USA; ^2^ Makerere University, Kampala, Central, Uganda; ^3^ University of North Carolina at Chapel Hill, Chapel Hill, NC, USA; ^4^ Makerere University, Kampala, Uganda

## Abstract

**Background:**

The population of adults aged 60 years and older in sub‐Saharan Africa (SSA) is projected to reach 670 million by 2030. The increasing number of older persons presents unique social and healthcare challenges, including dementia, which has not been well‐studied in rural SSA. This study we aimed to determine the prevalence and risk factors associated with dementia among older adults rural eastern Uganda.

**Method:**

A cross‐sectional study was conducted in rural eastern Uganda (Busia and Namayingo districts) from December 2023 to September 2024. Neurocognitive disorder in older persons was assessed using the Identification and Intervention for Dementia in Elderly Africans (IDEA) and Rowland University Dementia Assessment Scale (RUDAS) and a functional assessment tool (IDEA‐Instrumental Activities of Daily Living) tools. Depression was measured with the Patient Health Questionnaire‐9 and alcohol use disorder with CAGE scale. Frequencies and multivariable logistic regression analyses adjusted for relevant covariates were performed.

**Result:**

We enrolled 602 older persons (mean+/‐ standard deviation [SD] age 70.0+/‐9.0 years; 60.6% female; 39% with no formal education and 43% completed primary school; 48.5% widowed) and interviewed one caregiver each household. More than 65% of all older adults had elevated blood pressure and nearly 30% had a possible alcohol use disorder. More than half had depression and were socially isolated. We found 20.6% had possible dementia based on IDEA results. Multivariable regression showed age>=70 and social isolation and loneliness were risk factors for dementia, while completing primary school or greater and remunerated work were protective against dementia.

**Conclusion:**

There is a high prevalence of dementia among older adults in rural Uganda with older age, no formal education, social isolation and loneliness increasing risk of dementia. Targeted risk reduction of modifiable dementia risk factors exacerbated in rural resource‐limited settings are needed by developing public health programs for dementia awareness and risk factor modification in these regions.

